# Unraveling Online Mental Health Through the Lens of Early Maladaptive Schemas: AI-Enabled Content Analysis of Online Mental Health Communities

**DOI:** 10.2196/59524

**Published:** 2025-02-07

**Authors:** Beng Heng Ang, Sujatha Das Gollapalli, Mingzhe Du, See-Kiong Ng

**Affiliations:** 1 Integrative Sciences and Engineering Programme NUS Graduate School National University of Singapore Singapore Singapore; 2 Institute of Data Science National University of Singapore Singapore Singapore

**Keywords:** early maladaptive schemas, large language models, online mental health communities, case conceptualization, prompt engineering, artificial intelligence, AI

## Abstract

**Background:**

Early maladaptive schemas (EMSs) are pervasive, self-defeating patterns of thoughts and emotions underlying most mental health problems and are central in schema therapy. However, the characteristics of EMSs vary across demographics, and despite the growing use of online mental health communities (OMHCs), how EMSs manifest in these online support-seeking environments remains unclear. Understanding these characteristics could inform the design of more effective interventions powered by artificial intelligence to address online support seekers’ unique therapeutic needs.

**Objective:**

We aimed to uncover associations between EMSs and mental health problems within OMHCs and examine features of EMSs as they are reflected in OMHCs.

**Methods:**

We curated a dataset of 29,329 posts from widely accessed OMHCs, labeling each with relevant schemas and mental health problems. To identify associations, we conducted chi-square tests of independence and calculated odds ratios (ORs) with the dataset. In addition, we developed a novel group-level case conceptualization technique, leveraging GPT-4 to extract features of EMSs from OMHC texts across key schema therapy dimensions, such as *schema triggers* and *coping responses*.

**Results:**

Several associations were identified between EMSs and mental health problems, reflecting how EMSs manifest in online support-seeking contexts. Anxiety-related problems typically highlighted *vulnerability to harm or illness* (OR 5.64, 95% CI 5.34-5.96; *P*<.001), while depression-related problems emphasized unmet interpersonal needs, such as *social isolation* (OR 3.18, 95% CI 3.02-3.34; *P*<.001). Conversely, problems with eating disorders mostly exemplified negative self-perception and *emotional inhibition* (OR 1.89, 95% CI 1.45-2.46; *P*<.001). Personality disorders reflected themes of *subjugation* (OR 2.51, 95% CI 1.86-3.39; *P*<.001), while posttraumatic stress disorder problems involved distressing experiences and *mistrust* (OR 5.04, 95% CI 4.49-5.66; *P*<.001). Substance use disorder problems reflected negative self-perception of *failure to achieve* (OR 1.83, 95% CI 1.35-2.49; *P*<.001). Depression, personality disorders, and posttraumatic stress disorder were also associated with 12, 9, and 7 EMSs, respectively, emphasizing their complexities and the need for more comprehensive interventions. In contrast, anxiety, eating disorder, and substance use disorder were related to only 2 to 3 EMSs, suggesting that these problems are better addressed through targeted interventions. In addition, the EMS features extracted from our dataset averaged 13.27 (SD 3.05) negative features per schema, with 2.65 (SD 1.07) features per dimension, as supported by existing literature.

**Conclusions:**

We uncovered various associations between EMSs and mental health problems among online support seekers, highlighting the prominence of specific EMSs in each problem and the unique complexities of each problem in terms of EMSs. We also identified EMS features as expressed by support seekers in OMHCs, reinforcing the relevance of EMSs in these online support-seeking contexts. These insights are valuable for understanding how EMS are characterized in OMHCs and can inform the development of more effective artificial intelligence–powered tools to enhance support on these platforms.

## Introduction

### Background

Over recent years, mental health problems have become increasingly prevalent worldwide [1-3]. However, access to professional therapy remains limited for many individuals due to high treatment costs and a shortage of trained mental health professionals [4-6]. As a result, a growing number of individuals are seeking support for their problems through online mental health communities (OMHCs) [7-9]. These communities provide a more accessible alternative to professional therapy [10,11], allowing individuals to share their distressing experiences and receive peer support to help them cope [11,12]. These problems, typically shared through posts within OMHCs, not only reveal support seekers’ struggles but also reflect the underlying dysfunctional beliefs contributing to their distress. These insights are crucial in shaping more personalized and effective online interventions for support seekers [13,14].

A set of dysfunctional beliefs commonly present in OMHC posts is early maladaptive schemas (EMSs) [15,16]. Central to schema therapy (ST), EMSs are pervasive, self-defeating beliefs formed in childhood and often persist into adulthood. Young et al [15] identified 18 distinct types of EMSs (Table 1) that distort worldviews and are typically associated with mental health problems, such as anxiety [17-21]. Understanding these associations provides a more reliable method for anticipating potential schemas based on a mental health diagnosis, enabling proactive and targeted therapeutic interventions [15,22,23]. Similarly, recognizing the unique features of each schema (eg, “I’m incompetent when it comes to achievement,” corresponding to the *failure to achieve* schema) helps inform targeted interventions (eg, setting small, realistic goals to foster a sense of accomplishment). By addressing these schemas, these interventions can enhance therapy effectiveness and improve treatment outcomes.

However, the characteristics of these EMSs can vary across demographic groups. For example, Cámara and Calvete [18] identified a strong association between the *failure* schema and depression in Spanish university students. However, Wright et al [24] did not report such an association in their study of a US cohort. Similarly, Balsamo et al [25] did not observe a relationship between the *failure* schema and depression among young adults in Italy. Likewise, OMHCs likely present a demographically distinct population of online support seekers reflecting unique EMS characteristics [18,24-26]. Gaining a clearer understanding of these characteristics could provide valuable insights for policy making and the design of more effective digital therapeutic tools for this online support-seeking population. However, how EMSs are reflected in these communities remains largely unexplored, despite their growing importance in mental health support.

This research gap largely stems from the lack of tools for accurately identifying EMSs in the asynchronous, text-based environments of OMHCs. In professional practice, therapists use the Young Schema Questionnaire (YSQ) [27] to assess patients’ EMSs through clinically validated statements of dysfunctional beliefs, which patients rate on a scale from 1 (“completely untrue of me”) to 6 (“describes me perfectly”). Replicating this structured assessment in OMHCs is challenging due to the unstructured nature of online support-seeking interactions [15,28,29]. Overcoming this challenge requires interpreting EMSs directly from the text, which necessitates specialized natural language processing techniques to identify EMSs in OMHC posts. Recent advancements in large language models (LLMs) have demonstrated remarkable performance in complex language tasks, such as text classification, retrieval, and generation, which require nuanced interpretation of unstructured texts [30-32]. These models can capture subtle meanings in textual discourse, such as recognizing that the phrase “dark tunnel” in “I just feel like I’m stuck in a dark tunnel, and there’s no way out” metaphorically conveys despair, isolation, and hopelessness [33]. LLMs have also been used to predict mental health problems, such as depression, from online conversations [34-38] and have recently been applied to predict EMSs in OMHC posts [16].

**Table 1:** The taxonomy proposed by Young et al [15] of 18 early maladaptive schemas (EMSs) with representative examples from the Young Schema Questionnaire (YSQ) [27]. EMSs represent cognitive and emotional patterns that shape perception and behavior, often contributing to mental health problems.

EMS	Description	Examples from YSQ
Abandonment/instability	The perception that others will not be reliable enough to provide emotional support or connection, leading to a sense of instability and insecurity	“I worry that people I feel close to will leave me or abandon me.”
Approval seeking/recognition-seeking	A constant desire for approval, recognition, or acceptance from others to the extent that one compromises their authentic self	“Unless I get a lot of attention from others, I feel less important.”
Defectiveness/shame	A deep-seated belief that one is flawed, unlovable, or inferior, leading to feelings of shame and inadequacy	“I cannot understand how anyone could love me.”
Dependence/incompetence	A belief in one’s inability to handle daily responsibilities or make appropriate decisions independently	“I feel that I need someone I can rely on to give me advice about practical issues.”
Emotional deprivation	The belief that one’s emotional needs will not be adequately met by others	“For much of my life, I haven’t felt that I am special to someone.”
Emotional inhibition	The suppression or inhibition of emotions, often due to a fear of losing control or being overwhelmed by intense feelings	“People see me as uptight emotionally.”
Enmeshment/undeveloped self	An overly intense emotional attachment and proximity to one or more important individuals (typically parents), which hinders the process of becoming an independent individual and impedes normal social development	“I often found myself constantly turning to my parents for approval in every decision I made.”
Entitlement/grandiosity	An exaggerated sense of self-importance and a belief that one deserves special treatment	“I get very irritated when people won’t do what I ask of them.”
Failure to achieve	The belief that one has failed or will inevitably fail in achieving personal and practitioner goals, leading to a sense of underachievement	“I am humiliated by my failures and inadequacies in the work (or school) sphere.”
Insufficient self-control/self-discipline	An inability to control impulses, leading to difficulty in achieving long-term goals.	“I often do things impulsively that I later regret.”
Mistrust/abuse	A belief that others are likely to deceive, take advantage of, or harm the person emotionally or physically	“I have been physically, emotionally, or sexually abused by important people in my life.”
Negativity/pessimism	A pervasive focus on the negative aspects of life while neglecting the positive aspects	“People close to me consider me a worrier.”
Punitiveness	The belief that harsh punishment is deserved for perceived mistakes or failures	“I am a bad person who deserves to be punished.”
Self-sacrifice	The belief that one must meet the needs of others at the expense of one’s own needs and desires	“No matter how much I give, I feel it is never enough.”
Social isolation/alienation	The belief that one is different from others, leading to a sense of isolation and difficulty connecting with others	“I always feel on the outside of groups.”
Subjugation	A tendency to prioritize others’ needs and desires over one’s own because one feels coerced	“I let other people have their way, because I fear the consequences.”
Unrelenting standards/hypercriticalness	The belief that one must meet high standards of performance, usually to avoid criticism	“I can’t let myself off the hook easily or make excuses for my mistakes.”
Vulnerability to harm or illness	The belief that catastrophic events are imminent, leading to excessive worry and anxiety about potential harm or illness	“I feel that the world is a dangerous place.”

### Objectives

Using the EMS classifiers from the recent study on EMS prediction [16] and leveraging recent LLMs, we can identify EMS labels within large volumes of unstructured OMHC texts. This enables us to (1) uncover associations between EMSs and mental health problems within OMHCs and (2) examine how features of EMSs are reflected in online support-seeking populations. These insights will guide the development of online interventions powered by artificial intelligence (AI) and grounded in ST, tailored to meet the unique therapeutic needs of online support seekers [15].

## Methods

### Curating the Dataset and Identifying EMSs

We curated posts shared by support seekers in 3 highly frequented OMHCs, namely 7-Cups [39], Beyond-Blue [40], and Patient [41], from 2013 to November 2023. Using a custom Python-based web crawler developed for this study, we collected posts from subcommunities focusing on anxiety, depression, posttraumatic stress disorder (PTSD), personality disorders (PDs), eating disorders (EDs), and substance use disorders (SUDs). These mental health problems are prevalent among adults [42,43] and are often treated with ST [15].

To identify EMSs in OMHC posts, we used the entailment-based prediction model (EPM) from a recent study [16]. EPM predicts EMSs from a post by leveraging textual entailment, a sentence-level inference technique from natural language processing [16]. In textual entailment, a statement entails a hypothesis *h* if a human reader would infer that *h* is likely true based on *t* [44]. EPM computes the entailment between sentences in a post and statements from the YSQ. When a sentence entails a YSQ statement, the latter serves as an “explanation” for assigning the corresponding EMS label. For instance, the sentence “Since then I either try to find excuses not to ride or when I do try to ride, I have to push myself and have massive panic attacks.” entails the YSQ statement “I often feel that I’m going to have an anxiety attack.” This YSQ statement corresponds to the *vulnerability to harm or illness* schema, and the EPM labels the post with this schema. We used the Text-To-Text Transfer Transformer (Google; T5*-*LLM [45]) in EPM to compute entailment and assign EMS labels to each post.

The 18 EMSs in Table 1 are each represented as a binary categorical variable, where a value *1* indicates the relevance of a schema to an OMHC post, and *0* indicates nonrelevance. In a similar way, we labeled each post with 6 binary categorical variables, each corresponding to one of the mental health problems examined in this study.

### Uncovering Associations Between EMSs and Mental Health Problems

Using these categorical variables, we constructed 2×2 contingency tables (Table S1 in Multimedia Appendix 1) and calculated the expected frequency, E_ij_ (equation S1 in Multimedia Appendix 1), and the *χ^2^* statistic (equation S2 in Multimedia Appendix 1) for each pair of schema and mental health problem [46]. Each *χ^2^* value was compared to the critical value from the *χ^2^* distribution table with 1 *df* and a significance level of 0.05. For each *χ^2^* value that exceeded the critical value, we rejected the null hypothesis to indicate a statistically significant association between a schema and mental health problem. To quantify the strength of association for each statistically significant pair, we calculated the odds ratio (OR), OR_ems_mhp_ (equation S3 in Multimedia Appendix 1), and its 95% CI, OR_ems_mhp_95%CI_ (equation S4 in Multimedia Appendix 1) [47,48]. Following established guidelines for interpreting ORs [48], we considered an association inconclusive if the 95% CI of the OR included the null value of 1.

### Examining Features of EMSs With Group-Level Case Conceptualization

We further examined how online support seekers express their schemas by analyzing specific features reflected in their online posts. For this purpose, we used the case conceptualization technique, which is commonly used by therapists to identify key features related to patients’ beliefs and mental health problems, thus facilitating personalized therapeutic interventions. In ST, case conceptualization focuses on several dimensions, including *schema triggers* (situations that activate the schema), *emotions* (associated feelings), *negative thoughts* (irrational beliefs), *coping responses* (behaviors and reactions), and *bodily sensations* (physical and physiological experiences) [15].

We adapted this case conceptualization to our dataset using a data-driven AI approach, which we refer to as group-level case conceptualization. First, we prepared the data by using the EPM to identify sentences from OMHC posts that entailed specific YSQ statements, as outlined in the Curating the Dataset and Identifying EMSs section. These sentences reflected the dysfunctional beliefs and respective schemas established in the YSQ. We grouped these sentences according to their schema, resulting in a distribution of sentence counts by EMSs, which ranged from 8494 for *social isolation* to 98 for *subjugation*. To derive group-level features for each schema, we randomly sampled 10% of the sentences (in Multimedia Appendix 2, we demonstrate that the similarity scores between features extracted from different groups of randomly sampled sentences consistently average 0.79 (SD 0.09), which indicates substantial overlap in the features generated across groups. This validates the approach of conducting in-depth analysis on a single group of randomly sampled sentences for each schema and instructed GPT-4 (OpenAI; we used the model gpt-4-1106-preview for GPT-4) [32] through a custom prompt (Textbox 1) to extract common features among the sentences and categorize them along the dimensions of case conceptualization.

**Prompt used to instruct GPT-4 to extract common features from groups of randomly sampled sentences for each schema.**

Identify every common features exhibited from the given sentences. The features should belong to one of the following dimension (a) Schema Triggers (What are the situations that trigger the emotions, negative thoughts, coping responses, and bodily sensations?) (feature should start with: Situation where...), (b) Emotions (What are the feelings involved?) (feature should start with: Feeling...), (c) Negative thoughts (What irrational thoughts are going through the mind?) (feature should start with: Thinking that...), (d) Coping Responses (What are the behaviors and reactions?) (feature should start with: Responding by...), (e) Bodily Sensations (What are the physical and physiological experiences with the body?) (feature should start with: Experiencing...). Provide more than 1 relevant samples from the given sentences for each feature.

If a dimension has feature(s), the output should be in the format as this example:

(d) Coping Responses:

** Destructive or impulsive actions when upset.

• “When I’m angry or frustrated, I sometimes lose control of my emotions and destroy things.”

• “Im 22, and I have this problem when I get angry I can't control myself I yell hit stuff around me and often I hurt myself.”

If a dimension does not have any feature, the output should be in the format as this example:

(d) Coping Responses:

**NO_FEATURES.

Given sentences:

### Ethical Considerations

This study aligns with NUS-IRB Review Not Required guidelines by analyzing nonindividually identifiable data from OMHCs to explore the characteristics of EMSs. Specifically, we used textual content that was already anonymized through usernames and we further decoupled these pseudonyms and other identifying information from the data. In addition, the analysis focused exclusively on examining publicly available data, with no interactions with support seekers. This approach minimized the risks of introducing harmful or unsupportive content while also adhering to ethical guidelines and the responsible use of these online platforms [49,50].

## Results

### Dataset Statistics

Our dataset comprised 29,329 online posts in which support seekers shared their experiences with various mental health problems. Anxiety was the most frequently mentioned problem, appearing in 51.56% (15,149/20,329) of the posts, followed by depression, which appeared in 33.98% (9966/20,329) of the posts. PTSD, SUDs, PDs, and EDs correspondingly represented 8.75% (2565/20,329), 1.96% (576/20,329), 1.87% (548/20,329), and 1.79% (525/20,329) of the dataset. This distribution aligned with general sociodemographic trends in the prevalence of mental health problems. For example, anxiety is typically more prevalent than depression, while both are more common than PTSD, PDs, and EDs [42,51]. This suggests that the mental health problems discussed in OMHCs may reflect their real-world prevalence, suggesting that policies based on these trends could be effectively applied to online settings.

Regarding post length, the median sentence count was 10 (IQR 5-16). Word count showed similar variability, with a median of 214 words (IQR 124-356). This variability likely reflected the open nature of online support forums, where individuals can express themselves freely, unconstrained by the structured environments of in-person therapy. Unlike therapy sessions, where therapists guide conversations and encourage concise, focused responses, OMHCs allow support seekers to share their experiences through detailed, unfiltered narratives. Consequently, the dataset included both succinct and elaborate descriptions of mental distress.

As illustrated in Table 2, the distribution of EMSs, as predicted by EPM [16], was nonuniform across the dataset. Posts more frequently involved schemas related to *social isolation* and *vulnerability to harm or illness* than schemas such as *approval seeking* and *subjugation*. We hypothesize that this skewed distribution resulted from a combination of societal, environmental, biological, and psychological factors. For instance, modern societal pressures, pervasive social media use, and global crises, such as the COVID-19 pandemic, may amplify feelings of isolation and vulnerability [52,53]. In contrast, schemas such as *approval seeking* and *subjugation* may be less common due to cultural factors, including the emphasis on individualism and self-reliance in many societies [54].

**Table 2:** Distribution of early maladaptive schemas (EMSs) in posts on mental health problems shared by support seekers on 7-Cups, Beyond-Blue, and Patient, between 2013 and 2023 (N=29,329). The distribution is uneven, with certain schemas being more prevalent, reflecting the diverse emotional and cognitive patterns of individuals seeking support in online mental health communities.

EMSs	Posts with EMSs, n (%)
Social isolation/alienation	12820 (43.71)
Vulnerability to harm or illness	10191 (34.75)
Dependence/incompetence	5414 (18.46)
Abandonment/instability	3736 (12.74)
Enmeshment/undeveloped self	2942 (10.03)
Negativity/pessimism	2777 (9.47)
Defectiveness/shame	2451 (8.36)
Emotional inhibition	2006 (6.84)
Emotional deprivation	1916 (6.53)
Mistrust/abuse	1641 (5.6)
Insufficient self-control/self-discipline	1578 (5.38)
Self-sacrifice	1530 (5.22)
Failure to achieve	1316 (4.49)
Unrelenting standards/hypercriticalness	1301 (4.44)
Punitiveness	839 (2.86)
Entitlement/grandiosity	487 (1.66)
Approval seeking/recognition seeking	475 (1.62)
Subjugation	276 (0.94)

### Associations Between EMSs and Mental Health Problems

Among all mental health problems, depression had the highest number of statistically significant associations with 12 out of the 18 EMSs (refer to Table S1 in Multimedia Appendix 3 for specific *P* values, ORs, and 95% CI of these associations). This was followed by PDs and PTSD, which had 9 and 7 associations, respectively. In contrast, anxiety, EDs, and SUDs showed fewer associations, with only 2 to 3 EMSs associated with these problems.

In Table S1 in Multimedia Appendix 3, we present the strength of the association between specific schemas and particular mental health problems using ORs. Consistent with previous works [55,56], we used OR<1.5 to indicate small effect sizes (weak associations), OR<2.5 to indicate moderate effect sizes (moderate associations), OR<4.0 to indicate large effect sizes (strong associations), and OR>4.0 to indicate very large effect sizes (very strong associations). Higher ORs suggest stronger associations, indicating that certain EMSs are relatively more likely to be present in specific mental health problems observed in OMHCs compared to others.

For anxiety, the strongest association was observed with the *vulnerability to harm or illness* schema, reflecting a very large effect size. In contrast, *insufficient self-control* and *emotional inhibition* schemas exhibited weak associations with anxiety.

In the case of depression, schemas with strong associations included *social isolation*, *approval-seeking*, and *emotional deprivation*. Moderate associations were observed for *abandonment*, *defectiveness*, *dependence*, *failure to achieve*, and *negativity*. Meanwhile, *unrelenting standards*, *entitlement*, *punitiveness*, and *self-sacrifice* schemas showed weak associations.

For EDs, moderate associations were observed with the *emotional inhibition*, *defectiveness*, and *self-sacrifice* schemas.

In PDs, the *subjugation* schema exhibited a very strong association, followed by moderate associations for the *abandonment*, *entitlement*, *enmeshment*, *defectiveness*, *punitiveness*, and *mistrust* schemas. Weak associations were observed for *emotional deprivation* and *social isolation*.

PTSD showed a very strong association with the *mistrust* schema and strong associations with *punitiveness* and *subjugation*. The *emotional deprivation* schema demonstrated a moderate association with PTSD, while weak associations were observed for *enmeshment*, *self-sacrifice*, and *abandonment*.

For SUDs, a moderate association was observed with the *failure to achieve* schema, while the *dependence* schema exhibited a weak association.

### Features of EMSs Extracted From Group-Level Case Conceptualization

Features of each schema were identified using GPT-4, as detailed in Multimedia Appendix 4. On average, our group-level case conceptualization approach identified approximately 13 features per schema across the 5 dimensions of case conceptualization. *Schema triggers*, *emotions*, and *negative thoughts* each comprised approximately 3 features, followed by *coping responses* with about 2 features, and *bodily sensations* with 1 feature. Most features conveyed negative connotations. For instance, individuals with the *abandonment* schema often perceived themselves as burdens to others and frequently reported feelings of loneliness, anxiety, and emptiness.

These features also highlighted the unique characteristics of each schema. For instance, the *punitiveness* schema, which centers on self-blame, involved features, such as “situations where there is a fear of repeating past mistakes or events,” “feelings of guilt or shame over past actions or events,” “thinking that one cannot forgive oneself or others for past wrongdoings,” and “responding with self-harm or destructive behavior.” These features are elaborated on in the Discussion section.

## Discussion

### Principal Findings

#### Associations With EMSs Reflect Varying Complexities Among Mental Health Problems

The associations identified in this study highlight the varying complexities of mental health problems among online support-seeking populations. Depression exhibited numerous associations with EMSs, suggesting a considerable overlap with depression symptomatology. This indicates that EMSs may provide a valuable framework for understanding the diverse factors influencing the experience of depression, underscoring its multifaceted nature [57-60]. Similarly, the large number of associations between EMSs and either PDs or PTSD highlight the heterogeneous and complex nature of these mental health problems, emphasizing the need for personalized and comprehensive therapeutic approaches.

In contrast, fewer associations were observed between EMS and anxiety, EDs, and SUDs, respectively. This suggests more uniform schema-related underpinnings for these problems, suggesting that individuals with anxiety, EDs, or SUDs may present a more consistent and predictable symptomatology. Consequently, treatment strategies for these conditions could focus on targeting specific schema patterns rather than adopting broader, more comprehensive approaches.

#### The Prominence of “Vulnerability to Harm or Illness” in Anxiety Problems

The association observed between anxiety and *vulnerability to harm or illness* schema aligns with findings from previous studies, which have identified this schema in groups with higher anxiety levels, such as individuals affected by COVID-19 and college students [17,18,24,61,62]. The very strong association found in this study suggests that individuals seeking support for anxiety in OMHCs are very likely to experience and express heightened feelings of vulnerability. This observation aligns with the core purpose of OMHCs: creating a supportive safe space for individuals to share their challenges and vulnerabilities.

In contrast, associations between anxiety and the *insufficient self-control* and *emotional inhibition* schemas, though consistent with previous offline studies [62-69], were weak in OMHCs. One possible explanation is that individuals participating in OMHCs may exhibit greater self-discipline, as their active participation reflects motivation for change and growth [70]. In addition, the anonymity of OMHCs likely encourages more open disclosure, mitigating tendencies for emotional inhibition [12].

Overall, these findings suggest that anxiety-related problems discussed in OMHCs are prominently characterized by perceptions of vulnerability to harm and danger rather than deficits in self-control or emotional inhibition. This highlights the central role of vulnerability in the experiences of online support seekers with anxiety and underscores the importance of addressing these feelings in the development of online peer support interventions.

#### The Prominence of “Prolonged Unmet Interpersonal Needs” in Depression Problems

The associations between depression and schemas of *social isolation*, *approval seeking*, and *emotional deprivation* are consistent with previous research, suggesting that individuals with depression often experience social exclusion, seek external validation, and report unmet emotional needs [71-75]. These schemas showed strong associations in OMHCs, suggesting these schemas are more likely to appear and are central concerns for individuals seeking support for depression-related problems.

In contrast, schemas such as *abandonment*, *defectiveness*, *dependence*, *failure to achieve*, and *negativity* exhibited moderate associations, consistent with studies relating depression to struggles with fears of abandonment, low self-worth, reliance on others for emotional support, and pervasive pessimism [71,76-79]. These schemas are likely to appear in OMHC discussions on depression but may not dominate the discourse to the same extent as those with strong associations.

In contrast, schemas such as *unrelenting standards*, *entitlement*, *punitiveness*, and *self-sacrifice* demonstrated weak associations. These schemas are less likely to emerge in OMHC discourse on depression, although these schemas have been identified as relevant to depression in previous studies [74,77,79].

The varying strengths of association between these schemas indicate a focus on prolonged unmet interpersonal needs in depression-related discourse. Strongly associated schemas, such as *social isolation*, *approval seeking*, and *emotional deprivation*, highlight long-term difficulties in forming social and emotional connections. In addition, the *abandonment*, *defectiveness*, *dependence*, *failure to achieve*, and *negativity* schemas reflect interpersonal aspects, although seemingly primarily on the implications of unmet interpersonal needs. Indeed, depression is known to exacerbate interpersonal challenges, further intensifying fears of rejection, lowered self-esteem, interpersonal dependency, and dissatisfaction with both self and life [80-82]. In contrast, weakly associated schemas, such as *unrelenting standards* and *entitlement*, are less focused on unmet interpersonal needs and seem more related to self-directed or situational aspects of depression.

#### The Prominence of “Self-Neglect and Negative Self-Perception” in ED Problems

The association between the *emotional inhibition* schema and EDs aligns with previous research suggesting that individuals with disordered eating behaviors often use these behaviors to avoid confronting distressing emotions [83-85]. This avoidance hinders healthy emotional processing and could contribute to neglecting psychological well-being.

The *defectiveness* schema reflects deep-seated feelings of inadequacy and self-criticism, which are particularly prevalent in individuals with binge eating behaviors. These individuals often resort to binge eating as a coping mechanism to manage pervasive feelings of shame, self-criticism, and low self-worth [86-88].

The *self-sacrifice* schema reflects a tendency to prioritize others’ needs over one’s own well-being. This behavior is closely related to pathological altruism, which involves irrational behaviors aimed at promoting the welfare of others but with negative consequences for the individual [89]. In the context of EDs, previous studies have found that individuals engage in self-sacrifice driven by pathological altruism to cope with negative self-perception of inadequacy and defectiveness, often stemming from their maladaptive eating behaviors [90-93].

These schemas demonstrated moderate associations with EDs in OMHCs, suggesting that themes of self-neglect and negative self-perception are likely to emerge from the distress experienced by individuals seeking support for their EDs within these communities.

#### The Prominence of “Subjugation” in PD Problems

The strong association between the *subjugation* schema and PDs observed in this study aligns with findings from previous research on avoidant PD and borderline PD (BPD) [19,94,95]. This association suggests that individuals discussing PD-related challenges in OMHCs are more likely to articulate struggles related to feeling oppressed by others. These struggles are often accompanied by fears of retaliation, humiliation, or rejection when expressing their true feelings and needs [15]. These fears can hinder their ability to assert themselves, establish boundaries, and manage conflicts effectively.

Moderate associations were uncovered between PDs and schemas such as *abandonment*, *entitlement*, *defectiveness*, *mistrust*, *enmeshment*, and *punitiveness*. While these schemas likely appear in discussions about PDs, they are less prominent compared to the *subjugation* schema. The *abandonment*, *mistrust*, *defectiveness*, and *enmeshment* schemas are frequently observed in individuals with BPD [19,96-98], whereas *entitlement* is more frequently seen in individuals with narcissistic PD [99], and *punitiveness* is associated with both BPD and avoidant PD [19,96].

This study also found weak associations between PDs and schemas such as *emotional deprivation* and *social isolation*. While consistent with earlier studies on BPD [19,21,100], these findings suggest that these schemas are less emphasized in discussions about PDs within OMHCs.

Overall, themes of *subjugation* emerge as a central focus in PD problems discussed in OMHCs. These themes highlight patterns rooted in power dynamics, emotional oppression, and interpersonal dependency. They appear to overlap with other schemas, such as *abandonment*, *defectiveness*, *entitlement*, *mistrust*, *enmeshment*, and *punitiveness*. For instance, individuals who feel subjugated may develop a fear of abandonment if they challenge others’ expectations [15]. In contrast, schemas such as *emotional deprivation* and *social isolation* might be less prominent because they focus more on internal emotional states (eg, feelings of neglect or alienation) and less on the relational and power dynamics that are central to subjugation.

#### The Prominence of “Traumatizing Experiences” in PTSD Problems

The association between PTSD and the *mistrust* schema in OMHCs is consistent with research showing that individuals typically develop chronic mistrust following traumatic experiences involving betrayal, attachment disruptions, or abuse [101-106]. The very strong association suggests that mistrust is very likely to emerge from OMHC discussions on PTSD.

Similarly, the association between PTSD and the *punitiveness* schema highlights the prominent role of self-blame, which is frequently observed in individuals with PTSD [105,107-109]. The association with the *subjugation* schema indicates that individuals may express feelings of being controlled or oppressed, with the anonymity afforded by these online platforms likely encouraging the disclosure of such sensitive experiences [105,106,110,111]. These strong associations suggest that these schemas are more likely to appear in OMHC posts about PTSD.

The association between PTSD and the *emotional deprivation* schema aligns with findings from previous research on traumatized populations, such as veterans and survivors of interpersonal trauma [105,112]. The moderate association observed in this study suggests that emotional neglect is likely to be reflected in OMHC discussions on PTSD.

Consistent with previous studies that identified relationships between PTSD and schemas such as *enmeshment*, *self-sacrifice*, and *abandonment* [105,106], this study identified similar associations. However, the associations observed in OMHCs are comparatively weak, suggesting that these schemas are less central to PTSD experiences shared within these online platforms.

Overall, the findings suggest that individuals seeking PTSD support in OMHCs are more likely to describe acute traumatizing experiences, reflecting schemas such as *mistrust*, *punitiveness*, *subjugation*, and *emotional deprivation*. In contrast, schemas such as *enmeshment*, *self-sacrifice*, and *abandonment* play a lesser role in these discussions.

#### The Prominence of “Perceived Failure” in SUD Problems

The association between SUDs and the *failure to achieve* schema is consistent with previous research, suggesting that individuals who perceive themselves as underachievers are more susceptible to substance misuse, and vice versa [20,113-117]. The moderate association observed in OMHCs indicates that themes of inadequacy and failure are likely to emerge in posts related to SUDs.

In contrast, the weak association between the *dependence* schema and SUDs in OMHCs challenges the traditional understanding of the disorder, which often emphasizes a high degree of helplessness and complete reliance on others for daily functioning [118-122]. The weak association suggests that individuals with SUD seeking support in OMHCs may still retain some autonomy and self-sufficiency despite their struggles.

#### Extracted Features Reflect Known Characteristics of EMSs

The EMS features (features extracted by GPT-4 for each schema are as listed in Multimedia Appendix 4; the features referenced in this section are enclosed in quotes.) identified in OMHCs reflect the core characteristics of each schema and align with previous research. Notable examples are presented subsequently.

Several features of the *vulnerability to harm or illness* schema suggest that it is triggered when an individual “anticipates or experiences a panic attack,” “fears a negative health outcome,” “encounters social interactions or public places,” and “perceives everything is falling apart.” These are consistent with the findings of Young et al [15], who noted that this schema is often activated by fears of uncontrollable external events. Similarly, McGinn et al [123] found that heightened anxiety about bodily symptoms and low perceived control significantly increase vulnerability to panic. The looming vulnerability model by Riskind [124] provides additional support for this, proposing that perceived vulnerability amplifies anxiety, manifesting in hypervigilance and beliefs such as “something bad is going to happen” and “one’s health is in serious danger.” In addition, Faustino et al [17] related this schema to COVID-19–related anxiety, emphasizing how individuals with heightened vulnerability often interpret health concerns as severe. Dean et al [125] observed that social exclusion intensifies vulnerability, perceived harm, and avoidant behaviors, aligning with the feature of “avoiding situations or places.” In addition, features related to somatic symptoms, such as “physical discomfort or pain related to anxiety” and “heavy breathing or heart palpitations,” were identified, consistent with the findings of McGinn et al [123] on somatic responses to low perceived control and vulnerability. Another notable feature of this schema is “seeking reassurance or help.” Groves et al [126] found that patients awaiting medical diagnoses often sought reassurance from nurses, alleviating feelings of vulnerability and fostering greater optimism about medical outcomes.

Features of the *social isolation* schema indicate an increased sensitivity to “potential rejection,” often leading individuals to “self-isolate” and “avoid social interactions or places.” This behavior aligns with the findings of Watson and Nesdale [127] on how social rejection exacerbates isolation tendencies. Individuals with this schema often view themselves as “unworthy or unlovable,” believe “others are better off without them,” and believe they are “fundamentally different and unable to connect with others.” These beliefs mirror the low self-esteem associated with social isolation and exclusion, as highlighted by Stanley and Arora [128] and Verkuyten and Thijs [129]. Emotional struggles, such as feelings of “hopelessness or helplessness,” and at times, “fear or anxiety,” are also prevalent. Johari et al [130] reported similar themes among homeless youth in Iran, who frequently experienced loneliness, abandonment, and fears of social harassment. Despite these challenges, some individuals with this schema expressed a “desire for connection,” which is consistent with the observation by Cacioppo and Cacioppo [131] that loneliness, while heightening sensitivity to social threats, can also drive efforts to seek and rebuild social bonds.

The *emotional inhibition* schema is characterized by a persistent fear of having “personal issues or emotions disclosed,” particularly in “social interaction” settings. This is consistent with findings by Young et al [15] and Coggins and Fox [68], who noted that individuals with this schema often suppress emotions out of concern that “others will judge or misunderstand them.” As a result, they frequently experience feelings of being “overwhelmed by anxiety and worry,” “scared or fearful,” and at times, “helpless or hopeless.” This schema is commonly triggered in situations where “negative outcomes are anticipated,” as observed by Iwamitsu et al [132], who found that patients with breast tumor reported higher emotional distress when suppressing emotions after receiving their diagnosis. In addition, individuals with this schema often “avoid discussing personal issues” and “internalize feelings without seeking help.” These tendencies mirror the inhibition and withdrawal behaviors observed by Denollet and Duijndam [133] in adults who had been socially inhibited, highlighting an aversion to open emotional expression and a reluctance to seek support from others.

Individuals with the *mistrust* schema often exhibit behaviors such as being “untrusting or suspicious” and perceiving others as “deceitful or have malicious intent,” particularly when their “personal safety or well-being feels threatened.” This aligns with the findings by Young et al [15], who observed that individuals with this schema commonly view others as potential threats, making them feel vulnerable if they lower their guard. Similarly, LaMotte et al [104] found that mistrust plays a role in the relationship between betrayal in romantic relationships and subsequent experiences of intimate partner violence. Individuals with this schema often believe they “will always be a targeted or wronged,” aligning with the concept of victim sensitivity [134-136], which involves a heightened expectation of untrustworthy motives in others and intense reactions to perceived injustices. Pilkington et al [137] further highlighted the link between “mistrust” and accounts of violence, particularly in cases of domestic violence. Furthermore, those with this schema often “push others away” and prefer being “isolated or alone.” Smith and Rosen [138] observed similar patterns among older clients receiving methadone treatment, who tended to self-isolate because of their mistrust. This self-isolation undermines the development of healthy relationships that are essential for recovery.

The *failure to achieve* schema reflects a persistent belief that one is “not good enough” and has “ruined everything,” leading to cycles of “self-criticism.” Young et al [15] similarly noted that individuals with this schema often feel doomed to failure in both present and future endeavors. Wood et al [139] found that such individuals frequently engage in social comparison, seeking reassurance by comparing themselves to others they perceive as less successful. This aligns with the observation that individuals often “compare themselves to peers or societal standards.” However, comparisons with those perceived as more successful can often intensify feelings of “jealousy,” “disappointment in themselves,” and “helplessness,” sometimes causing them to “give up trying” to improve their circumstances. Leahy [140] observed that unfavorable comparisons in competitive environments can lead to envy, humiliation, helplessness, and withdrawal. This withdrawal reinforces feelings of failure, perpetuating the belief in one’s inability to succeed and deepening cycles of self-criticism.

### Limitations

This study focused exclusively on 3 widely accessed OMHCs that use English as the primary mode of communication. To broaden the cultural and linguistic diversity of the sample, future research could explore OMHCs that use other languages and compare the findings of this study with subsequent research to verify whether our results extend to OMHCs in different sociolinguistic and cultural contexts, where schema expression may vary. In addition, while the anonymity of these platforms encourages openness, it limits access to sociodemographic data (eg, age, gender, and socioeconomic status), which might be important in shaping mental health perspectives and schema characteristics. Future studies could explore methods that allow for the ethical anonymous collection of demographic data or use validated techniques to infer demographic characteristics while maintaining user privacy, which is of utmost importance. These suggestions are essential for assessing the generalizability of the EMS characteristics uncovered in this study. Expanding the demographic and cultural scope in future research would help validate and deepen these findings, contributing to a more comprehensive understanding of EMSs in online support-seeking environments.

### Conclusions

In this study, we examined the characteristics of EMSs within OMHCs, situating our findings within the existing literature. By analyzing a large dataset of posts from widely accessed OMHCs, annotated with EMS labels using a state-of-the-art EMS classifier, we identified key EMS features and various associations between EMSs and mental health problems. Notably, we found that depression, PDs, and PTSD were associated with a wide range of EMSs, highlighting the complexity of these multifaceted problems. These findings suggest that interventions for these problems could benefit from addressing multiple EMSs simultaneously. In contrast, mental health problems with fewer EMS associations, such as anxiety, may respond more effectively to more targeted interventions focused on specific schemas. In addition, we identified EMSs as more prominently associated with certain mental health problems in OMHCs. For example, anxiety-related posts typically center around vulnerability to harm, while depression-related posts reflect prolonged unmet interpersonal needs. Negative self-perception and self-neglect are prevalent in posts about EDs, while posts about PDs involve themes of subjugation. Posts about PTSD often describe traumatic experiences, and those concerning SUD are marked by themes of personal failure. A key contribution of this study is the novel group-level case conceptualization technique, which uses recent advanced LLMs and prompt engineering techniques to extract EMS features from OMHC posts. This approach provides valuable insights into how EMSs are expressed in online support-seeking environments and has the potential to inform personalized, ST-focused interventions. Overall, this study provides a foundational understanding of EMS characteristics in online support-seeking contexts and their role in shaping mental health experiences. Our findings highlight the potential for tailored, ST-based interventions in online peer support settings, which could enhance therapeutic outcomes and promote the mental well-being of individuals seeking support in these online communities.

## Appendix

Multimedia Appendix 1 Statistical approach for uncovering associations.

Multimedia Appendix 2 Consistency of the extracted features.

Multimedia Appendix 3 Associations between early maladaptive schemas and mental health problems.

Multimedia Appendix 4 Features of early maladaptive schemas.

## Abbreviations

AI: artificial intelligence
BPD: borderline personality disorder
ED: eating disorder
EMS: early maladaptive schema
EPM: entailment-based prediction model
LLM: large language model
OMHC: online mental health community
OR: odds ratio
PD: personality disorder
PTSD: posttraumatic stress disorder
ST: schema therapy
SUD: substance use disorder
YSQ: Young Schema Questionnaire

## Data Availability

The datasets generated and analyzed during this study are available from the corresponding author on reasonable request.
