# Supplementary material for: Unraveling Online Mental Health Through the Lens of Early Maladaptive Schemas: AI-Enabled Content Analysis of Online Mental Health Communities
Source: J Med Internet Res. 2025 Feb 7;27:e59524. doi: 10.2196/59524 (PMC11845891; doi:10.2196/59524)
Supplement: Multimedia Appendix 1 [file jmir_v27i1e59524_app1.doc]

# Multimedia Appendix 1: Statistical Approach for Uncovering Associations

Table S1. Example of a 2x2 contingency table showing the observed frequencies (cells A, B, C, and D) between the binary categorical variables “Depression” and “Abandonment” schema.

|  | **Abandonment** **1 (YES)** | **Abandonment** **0 (NO)** |
| --- | --- | --- |
|  |  |  |
| **Depression 1 (YES)** |  |  |
|  | A | B |
| **Depression 0 (NO)** |  |  |
|  | C | D |

E_ij_ = (n_i_ x n_j_) / n **(S1)**

where:

- E_ij_ is the expected frequency for cell in row i and column j of 2x2 contingency table (Table S1)
- n_i_ is the total of row i
- n_j_ is the total of column j
- n is the grand total

χ^2^ = Σ ((O_ij_ – E_ij_)^2^ / E_ij_) **(S2)**

where:

- χ^2^ is the Chi-square statistic
- O_ij_ is the observed frequency for cell in row i and column j of the 2x2 contingency table
- E_ij_ is the expected frequency for cell in row i and column j of the 2x2 contingency table

ORems_mhp = (A/B) / (C/D) **(S3)**

where:

- ORems_mhp is the Odds Ratio indicating the strength of an association between an EMS and mental health problem
- A is the observed frequency where both target EMS and mental health problem are involved (Table S1)
- B is the observed frequency where target EMS is not involved but mental health problem is involved
- C is the observed frequency where target EMS is involved but mental health problem is not involved
- D is the observed frequency where both target EMS and mental health problem are not involved

ORems_mhp_95%CI =e^ln(OR)±1.96sqrt(1/A+1/B​+1/C​+1/D)^ **(S4)**​​

where:

- ORems_mhp_95%CI is the Odds Ratio 95% CI
- ln(OR) is the natural logarithm of the ORems_mhp (Equation S3)
- A, B, C and D are similarly defined in (Equation S3)
