# Supplementary material for: Unraveling Online Mental Health Through the Lens of Early Maladaptive Schemas: AI-Enabled Content Analysis of Online Mental Health Communities
Source: J Med Internet Res. 2025 Feb 7;27:e59524. doi: 10.2196/59524 (PMC11845891; doi:10.2196/59524)
Supplement: Multimedia Appendix 2 [file jmir_v27i1e59524_app2.doc]

# Multimedia Appendix 2: Consistency of the Extracted Features

## Methods:

To evaluate the consistency of features extracted by our group-level case conceptualization approach and validate the robustness of the method, we conducted repeated experiments for each schema. Specifically, we curated 10 groups of randomly sampled sentences relevant to each schema. GPT-4 was then instructed with a specially crafted prompt (Textbox 1) to extract features for each group along the dimensions of case conceptualization. The extracted features were encoded into high-dimensional vectors using the pre-trained sentence-transformer, “all-distilroberta-v1” [1]. Pairwise cosine similarity scores were calculated between feature vectors from different groups to quantify semantic alignment. These scores were averaged for each schema and dimension, yielding a mean similarity score representing overall semantic consistency. Higher scores reflected greater consistency in extracted features across groups for a schema and dimension, validating the robustness of our approach despite variability introduced by random subsampling.

## Findings:

The results demonstrate strong consistency in the features identified across randomly sampled sentence groups for most schemas and dimensions. As illustrated in (Figure S1), features for the “Subjugation” schema along the “Coping Responses” dimension yielded a high average pairwise cosine similarity score of 0.83 (SD 0.05). Similar patterns were observed across other schemas and dimensions, with similarity scores typically averaging around 0.8 (SD 0.05) (see Table S1). These findings indicate that the features extracted for most schemas and dimensions are reliably consistent across sentence groups. However, lower average similarity scores (below 0.6) were observed in a few cases, particularly those involving the “Bodily Sensations” dimension. A qualitative analysis revealed that these lower scores were attributable to certain sentence groups containing insufficient descriptions of bodily sensations, which constrained GPT-4’s ability to extract relevant features for this dimension.


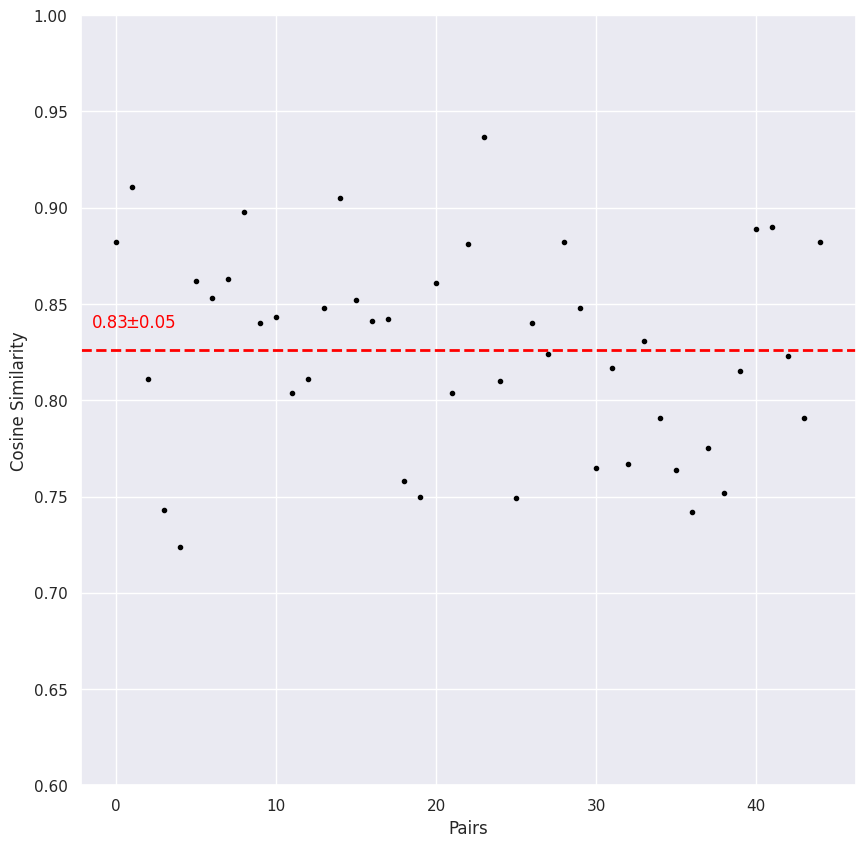


Figure S1. Scatterplot of pairwise cosine similarity scores for the “Abandonment/instability” schema in the “Coping Responses” dimension. Each point on this plot represents a similarity score for a pairing between two of the 10 independent groups for this schema and dimension, illustrating the consistency of features extracted across different groups.

Table S1. Mean and standard deviation of cosine similarity scores for features extracted across each schema and dimension. The scores demonstrate a high degree of semantic consistency in the features identified across most schemas and dimensions. However, a few schemas within the “Bodily Sensations” dimension exhibited notably lower mean cosine similarity scores.

| **Schema** | **Dimension** | | | | |
| --- | --- | --- | --- | --- | --- |
|  | Schema Triggers | Emotions | Negative Thoughts | Coping Responses | Bodily Sensations |
|  |  |  |  |  |  |
|  |  |  |  |  |  |
| Abandonment/  Instability | 0.840 ±0.04 | 0.798 ±0.08 | 0.801  ±0.05 | 0.826  ±0.05 | 0.813  ±0.04 |
| Approval-  Seeking/  Recognition-  Seeking | 0.833  ±0.04 | 0.814  ±0.07 | 0.814  ±0.05 | 0.833  ±0.05 | 1.00  ±0.00 |
| Defectiveness/  Shame | 0.812  ±0.04 | 0.869  ±0.04 | 0.837  ±0.04 | 0.868  ±0.02 | 0.824  ±0.04 |
| Dependence/  Incompetence | 0.822  ±0.06 | 0.862  ±0.05 | 0.863  ±0.03 | 0.857  ±0.04 | 0.718  ±0.37 |
| Emotional Deprivation | 0.856  ±0.03 | 0.849  ±0.04 | 0.809  ±0.04 | 0.839  ±0.04 | 0.483  ±0.35 |
| Emotional Inhibition | 0.868  ±0.04 | 0.846  ±0.03 | 0.804  ±0.04 | 0.842  ±0.04 | 0.815  ±0.03 |
| Enmeshment/  Undeveloped Self | 0.827  ±0.04 | 0.830  ±0.06 | 0.820  ±0.04 | 0.839  ±0.03 | 0.813  ±0.04 |
| Entitlement/  Grandiosity | 0.832  ±0.04 | 0.765  ±0.06 | 0.792  ±0.04 | 0.834  ±0.03 | 0.529  ±0.23 |
| Failure to  Achieve | 0.832  ±0.05 | 0.819  ±0.05 | 0.811  ±0.05 | 0.444  ±0.38 | 0.533  ±0.46 |
| Insufficient  Self-Control/  Self-Discipline | 0.659  ±0.09 | 0.739  ±0.07 | 0.728  ±0.10 | 0.819  ±0.10 | 0.820  ±0.06 |
| Mistrust/ Abuse | 0.827  ±0.04 | 0.820  ±0.07 | 0.834  ±0.05 | 0.843  ±0.03 | 0.527  ±0.33 |
| Negativity/  Pessimism | 0.805  ±0.05 | 0.767  ±0.07 | 0.835  ±0.05 | 0.806  ±0.06 | 0.760  ±0.09 |
| Punitiveness | 0.814  ±0.04 | 0.803  ±0.07 | 0.778  ±0.06 | 0.815  ±0.04 | 0.595  ±0.26 |
| Self-Sacrifice | 0.824  ±0.04 | 0.836  ±0.03 | 0.833  ±0.04 | 0.839  ±0.05 | 0.549  ±0.23 |
| Social Isolation/ Alienation | 0.755  ±0.08 | 0.818  ±0.07 | 0.771  ±0.10 | 0.813  ±0.07 | 0.643  ±0.28 |
| Subjugation | 0.784  ±0.06 | 0.682  ±0.09 | 0.738  ±0.07 | 0.751  ±0.05 | 0.684  ±0.41 |
| Unrelenting Standards/ Hyper-criticalness | 0.820  ±0.05 | 0.797  ±0.05 | 0.763  ±0.04 | 0.807  ±0.04 | 0.817  ±0.04 |
| Vulnerability to  Harm or Illness | 0.768  ±0.07 | 0.858  ±0.05 | 0.808  ±0.05 | 0.812  ±0.05 | 0.842  ±0.03 |

## Discussions:

The findings demonstrate a high level of semantic consistency in the features identified across schemas and dimensions, even when derived from independent groups of randomly sampled sentences. This strong consistency validates random subsampling as a practical and reliable method for selecting representative subsets of sentences associated with each schema. Also, random subsampling approximately 10% of the sentences for each schema appears sufficient for consistent and reliable feature extraction, supporting the feasibility of this approach for group-level case conceptualization.

However, a few schemas within the “Bodily Sensations” dimension (e.g. Self-Sacrifice) showed notably lower average cosine similarity scores (below 0.6) compared to the typical scores of around 0.8. This likely reflects the scarcity of explicit discussions about bodily sensations in online mental health discourse, limiting GPT-4’s ability to consistently identify features in this dimension.

These results highlight both the strengths and limitations of the proposed group-level case conceptualization approach for schema-based analyses in online mental health contexts. While random subsampling is generally effective, dimensions or schemas with sparse data may require additional strategies, such as augmenting input data with similar examples or refining prompts, to ensure more consistent feature extraction. Future research should explore these strategies to address the challenges observed in underrepresented dimensions like “Bodily Sensations”.

## References:

1. Reimers N, Gurevych I, editors. Sentence-BERT: Sentence Embeddings using Siamese BERT-Networks. Proceedings of the 2019 Conference on Empirical Methods in Natural Language Processing and the 9th International Joint Conference on Natural Language Processing (EMNLP-IJCNLP); 2019.
