# Supplementary material for: Unraveling Online Mental Health Through the Lens of Early Maladaptive Schemas: AI-Enabled Content Analysis of Online Mental Health Communities
Source: J Med Internet Res. 2025 Feb 7;27:e59524. doi: 10.2196/59524 (PMC11845891; doi:10.2196/59524)
Supplement: Multimedia Appendix 3 [file jmir_v27i1e59524_app3.doc]

# Multimedia Appendix 3: Associations between Early Maladaptive Schemas and Mental Health Problems

**Table S1.** Odds ratios (ORs) and 95% CI for significant associations (*P*<.05) between early maladaptive schemas (EMSs) and mental health problems, as identified by the chi-square test of independence. Depression, personality disorders (PDs), and posttraumatic stress disorder (PTSD) showed more associations with EMSs compared to anxiety, eating disorders (EDs), and substance use disorders (SUDs). The strength of associations ranged from weak (OR<1.5) to moderate (OR<2.5), strong (OR<4.0), and very strong (OR>4.0).

| EMS | Anxiety | | Depression | | ED | | PD | | PTSD | | SUD | |
| --- | --- | --- | --- | --- | --- | --- | --- | --- | --- | --- | --- | --- |
|  | OR (95% CI) | *P*-value | OR (95% CI) | *P*-value | OR (95% CI) | *P*-value | OR (95% CI) | *P*-value | OR (95% CI) | *P*-value | OR (95% CI) | *P*-value |
|  | | | | | | | | | | | | |
| Abandonment/instability | —^a^ | — | 2.19 (2.05-2.35) | <.001 | — | — | 1.98 (1.59-2.39) | <.001 | 1.19 (1.06-1.33) | .009 | — | — |
| Approval seeking/recognition seeking | — | — | 2.7 (2.28-3.29) | <.001 | — | — | — | — | — | — | — | — |
| Defectiveness/shame | — | — | 2.38 (2.19-2.59) | <.001 | 1.85 (1.44-2.37) | <.001 | 1.73 (1.35-2.22) | <.001 | — | — | — | — |
| Dependence/incompetence | — | — | 1.65 (1.55-1.75) | <.001 | — | — | — | — | — | — | 1.47 (1.22-1.78) | <.001 |
| Emotional deprivation | — | — | 2.50 (2.28-2.75) | <.001 | — | — | 1.48 (1.10-1.98) | .01 | 1.94 (1.70-2.21) | <.001 | — | — |
| Emotional inhibition | 1.14 (1.04-1.25) | .02 | — | — | 1.89 (1.45-2.46) | <.001 | — | — | — | — | — | — |
| Enmeshment/undeveloped self | — | — | — | — | — | — | 1.81 (1.44-2.27) | <.001 | 1.48 (1.32-1.67) | <.001 | — | — |
| Entitlement/grandiosity | — | — | 1.34 (1.12-1.60) | .02 | — | — | 1.93 (1.18-3.14) | .02 | — | — | — | — |
| Failure to achieve | — | — | 2.21 (1.99-2.48) | <.001 | — | — | — | — | — | — | 1.83 (1.35-2.49) | <.001 |
| Insufficient self-control/self-discipline | 1.47 (1.32-1.63) | <.001 | — | — | — | — | — | — | — | — | — | — |
| Mistrust/abuse | — | — | — | — | — | — | 1.56 (1.15-2.12) | .006 | 5.04 (4.49-5.66) | <.001 | — | — |
| Negativity/pessimism | — | — | 1.69 (1.57-1.84) | <.001 | — | — | — | — | — | — | — | — |
| Punitiveness | — | — | 1.24 (1.08-1.43) | .02 | — | — | 1.57 (1.03-2.38) | .049 | 2.59 (2.18-3.10) | <.001 | — | — |
| Self-sacrifice | — | — | 1.23 (1.11-1.37) | .001 | 1.60 (1.16-2.19) | .006 | — | — | 1.43 (1.21-1.68) | <.001 | — | — |
| Social isolation/alienation | — | — | 3.18 (3.02-3.34) | <.001 | — | — | 1.45 (1.22-1.71) | <.001 | — | — | — | — |
| Subjugation | — | — | — | — | — | — | 4.22 (2.66-6.69) | <.001 | 2.51 (1.86-3.39) | <.001 | — | — |
| Unrelenting standards/hypercriticalness | — | — | 1.44 (1.29-1.61) | <.001 | — | — | — | — | — | — | — | — |
| Vulnerability to harm or illness | 5.64 (5.34-5.96) | <.001 | — | — | — | — | — | — | — | — | — | — |

^a^Not applicable.
