# Supplementary material for: Unraveling Online Mental Health Through the Lens of Early Maladaptive Schemas: AI-Enabled Content Analysis of Online Mental Health Communities
Source: J Med Internet Res. 2025 Feb 7;27:e59524. doi: 10.2196/59524 (PMC11845891; doi:10.2196/59524)
Supplement: Multimedia Appendix 4 [file jmir_v27i1e59524_app4.doc]

# Multimedia Appendix 4: Features of Early Maladaptive Schemas

Textbox S1. Features of Abandonment/Instability schema identified by GPT-4 (Textbox 1) and group of randomly sampled sentences for the schema.

| Schema Triggers (Situation where …):   - one experiences a significant loss or change - one fears abandonment or rejection - one feels a lack of support or connection   Emotions (Feeling …):   - alone or isolated - anxious or scared - empty or hopeless   Negative Thoughts (Thinking that …):   - one is a burden to others - one fears abandonment or rejection - one feels a lack of support or connection   Coping Responses (Responding by …):   - avoiding social interactions - isolating oneself - seeking reassurance   Bodily Sensations (Experiencing …):   - Physical symptoms of anxiety - Experiencing symptoms of depression |
| --- |

Textbox S2. Features of Approval-Seeking/Recognition-Seeking schema identified by GPT-4 (Textbox 1) and group of randomly sampled sentences for the schema.

| Schema Triggers (Situation where …):   - one’s self-worth or identity is challenged - one’s social standing or inclusion is in question   Emotions (Feeling …):   - hurt or disappointed - lonely or isolated   Negative Thoughts (Thinking that …):   - one is a burden or unworthy - one is not valued or loved   Coping Responses (Responding by …):   - altering one’s behavior to fit in or be accepted - seeking validation or attention   Bodily Sensations (Experiencing …):   - - |
| --- |

Textbox S3. Features of Defectiveness/Shame schema identified by GPT-4 (Textbox 1) and group of randomly sampled sentences for the schema.

| Schema Triggers (Situation where …):   - one anticipates or experiences rejection - one feels misunderstood or alone - one is contemplating sharing their personal struggles - one is faced with their own self-image - one perceives themselves negatively in social contexts   Emotions (Feeling …):   - fear or anxiety about one’s own emotions or reaction - hopelessness or helplessness - self-hatred or self-disgust - unloved or unwanted   Negative Thoughts (Thinking that …):   - one is inherently flawed or defective - one is unworthy of love or friendship - one’s existence is pointless or that they are a burden - others dislike or hate them   Coping Responses (Responding by …):   - avoiding social interactions or fearing them - hiding true feelings or putting on a façade - not sharing personal struggles or keeping secrets   Bodily Sensations (Experiencing …):   - - |
| --- |

Textbox S4. Features of Dependence/Incompetence schema identified by GPT-4 (Textbox 1) and group of randomly sampled sentences for the schema.

| Schema Triggers (Situation where …):   - daily responsibilities feel overwhelming - interpersonal relationships are challenging - self-sufficiency is compromised - support is perceived as inadequate despite availability   Emotions (Feeling …):   - anxious - depressed - helpless or hopeless - overwhelmed   Negative Thoughts (Thinking that …):   - one is a burden - one is incapable or inadequate - one’s efforts are futile   Coping Responses (Responding by …):   - expressing a desire to give up - isolating oneself - seeking help or support   Bodily Sensations (Experiencing …):   - physical symptoms of anxiety - sensations of intense emotion |
| --- |

Textbox S5. Features of Emotional Deprivation schema identified by GPT-4 (Textbox 1) and group of randomly sampled sentences for the schema.

| Schema Triggers (Situation where …):   - individual feels isolated or lacks support - individual has a history of negative interpersonal relationships - individual’s efforts to seek help or support are dismissed or invalidated   Emotions (Feeling …):   - loneliness or abandonment - worthlessness or low self-esteem - unappreciated or unloved   Negative Thoughts (Thinking that …):   - one is undeserving of love or success - one’s problems are insignificant or that they should be able to cope alone   Coping Responses (Responding by …):   - ending relationships or avoiding connections due to fear of being a burden - isolating oneself or not seeking help   Bodily Sensations (Experiencing …):   - - |
| --- |

Textbox S6. Features of Emotional Inhibition schema identified by GPT-4 (Textbox 1) and group of randomly sampled sentences for the schema.

| Schema Triggers (Situation where …):   - loss of control is perceived or feared - negative outcomes are anticipated - personal issues or emotions might be disclosed - social interaction is required   Emotions (Feeling …):   - embarrassed or ashamed - helpless or hopeless - overwhelmed by anxiety and worry - scared or fearful   Negative Thoughts (Thinking that …):   - one is unable to cope with situations - one’s emotions or reactions are not valid or are a burden to others - others will judge or misunderstand   Coping Responses (Responding by …):   - avoiding discussing personal issues - internalizing feelings and not seeking help   Bodily Sensations (Experiencing …):   - increased heart rate and fear of passing out - physical symptoms of anxiety such as dizziness or shaking |
| --- |

Textbox S7. Features of Enmeshment/Undeveloped Self schema identified by GPT-4 (Textbox 1) and group of randomly sampled sentences for the schema.

| Schema Triggers (Situation where …):   - one anticipates or experiences abandonment or rejection - one is in social settings or around people - one’s independence or self-sufficiency is challenged - one’s safety or security feels threatened   Emotions (Feeling …):   - anxious or nervous - judged or insecure - overwhelmed or unable to cope - scared or terrified   Negative Thoughts (Thinking that …):   - one is in constant danger or at risk - one is inadequate or will fail - one is responsible for everything - one will be abandoned or left alone   Coping Responses (Responding by …):   - avoiding social interactions or relationships - expressing difficulty in controlling emotions - isolating oneself or withdrawing from activities - seeking constant reassurance or checking on loved ones   Bodily Sensations (Experiencing …):   - hypervigilance or heightened alertness - physical discomfort or symptoms in social situations - symptoms of anxiety or panic |
| --- |

Textbox S8. Features of Entitlement/Grandiosity schema identified by GPT-4 (Textbox 1) and group of randomly sampled sentences for the schema.

| Schema Triggers (Situation where …):   - confrontation is possible or anticipated - expectations or ambitions are not met - one feels misunderstood or judged by others - personal autonomy is challenged or restricted   Emotions (Feeling …):   - anger - being upset - fear - frustration - of hate   Negative Thoughts (Thinking that …):   - one is a burden - one is being held back by oneself - one is missing out on life due to societal obligations - one is not living up to their potential   Coping Responses (Responding by …):   - avoiding situations or people - expressing anger or frustration   Bodily Sensations (Experiencing …):   - physical manifestations of anger |
| --- |

Textbox S9. Features of Failure to Achieve schema identified by GPT-4 (Textbox 1) and group of randomly sampled sentences for the schema.

| Schema Triggers (Situation where …):   - one compares oneself to peers or societal standards - one experiences personal setbacks or failures - one perceives others as more successful or happier   Emotions (Feeling …):   - disappointed in oneself - jealous - like a failure - lost and hopeless   Negative Thoughts (Thinking that …):   - one has ruined everything - one is a failure - one is not good enough   Coping Responses (Responding by …):   - engaging in self-criticism - giving up or ceasing to try   Bodily Sensations (Experiencing …):   - - |
| --- |

Textbox S10. Features of Insufficient Self-Control/Self-Discipline schema identified by GPT-4 (Textbox 1) and group of randomly sampled sentences for the schema.

| Schema Triggers (Situation where …):   - one feels overwhelmed or challenged by daily tasks - one is faced with potential failure or defeat   Emotions (Feeling …):   - angry or irritable - anxious or panicked   Negative Thoughts (Thinking that …):   - one is doomed to fail or not succeed - one is incapable or inadequate   Coping Responses (Responding by …):   - avoiding or procrastinating - engaging in destructive or impulsive actions   Bodily Sensations (Experiencing …):   - physical symptoms like tremors or heart palpitations - sensations of restlessness or inability to relax |
| --- |

Textbox S11. Features of Mistrust/Abuse schema identified by GPT-4 (Textbox 1) and group of randomly sampled sentences for the schema.

| Schema Triggers (Situation where …):   - past trauma is reminiscent or triggered - personal safety or well-being feels threatened - social interaction is involved - trust is required or expected   Emotions (Feeling …):   - fear or paranoia - isolated or alone - untrusting or suspicious - vulnerable or exposed   Negative Thoughts (Thinking that …):   - one is unworthy or inadequate - one will always be a victim - others are deceitful or have malicious intent   Coping Responses (Responding by …):   - avoiding or isolating oneself - pushing others away - seeking control over one’s environment or self   Bodily Sensations (Experiencing …):   - heightened alertness or vigilance - physical symptoms of anxiety or fear |
| --- |

Textbox S12. Features of Negativity/Pessimism schema identified by GPT-4 (Textbox 1) and group of randomly sampled sentences for the schema.

| Schema Triggers (Situation where …):   - one anticipates negative outcomes or failure - one experiences fluctuations in mood or well-being - one’s anxiety affects interpersonal relationship - one’s efforts seem futile or are not recognized   Emotions (Feeling …):   - afraid or phobic - anxious or worried - overwhelmed or hopeless - sad or depressed   Negative Thoughts (Thinking that …):   - one is a burden or negative influence on others - one’s emotional state is permanent or unchangeable - things will never improve   Coping Responses (Responding by …):   - self-harming or engaging in destructive behavior - withdrawing or avoiding social interactions   Bodily Sensations (Experiencing …):   - fluctuations in physical well-being - physical symptoms of anxiety or stress |
| --- |

Textbox S13. Features of Punitiveness schema identified by GPT-4 (Textbox 1) and group of randomly sampled sentences for the schema.

| Schema Triggers (Situation where …):   - one is reminded of or confronted with past abuse or mistreatment - past behaviors or events are recalled - there is a fear of repeating past mistakes or events   Emotions (Feeling …):   - anger or frustration towards oneself or others - fear or anxiety about the future or potential repetition of past events - guilt or shame over past actions or events   Negative Thoughts (Thinking that …):   - one cannot forgive oneself or others for past wrongdoings - one is to blame for past events or situations - one is worthless or deserves punishment   Coping Responses (Responding by …):   - avoidance or withdrawal from situations - self-harm or destructive behavior - self-punishment or self-criticism   Bodily Sensations (Experiencing …):   - physical urges to self-harm or engage in violence when upset - sensations of misery or self-hatred |
| --- |

Textbox S14. Features of Self-Sacrifice schema identified by GPT-4 (Textbox 1) and group of randomly sampled sentences for the schema.

| Schema Triggers (Situation where …):   - one fears negative outcomes from seeking help - one feels responsible for others’ happiness or well-being - one perceives themselves as a burden or inadequate   Emotions (Feeling …):   - fear or anxiety - guilt or shame - overwhelmed or helpless   Negative Thoughts (Thinking that …):   - one is not worthy of help or support - one’s presence or actions are a burden to others   Coping Responses (Responding by …):   - isolating oneself or withdrawing from social interactions - prioritizing others’ needs over one’s own   Bodily Sensations (Experiencing …):   - physical symptoms of anxiety or stress |
| --- |

Textbox S15. Features of Social Isolation/Alienation schema identified by GPT-4 (Textbox 1) and group of randomly sampled sentences for the schema.

| Schema Triggers (Situation where …):   - evoke a sense of isolation or abandonment - involve social interactions or potential rejection - trigger fears of illness or crisis when alone   Emotions (Feeling …):   - alone or abandoned - empty or numb - hopeless or helpless - scared or anxious   Negative Thoughts (Thinking that …):   - one is unworthy or unlovable - others are better off without them - they are fundamentally different and cannot connect with others   Coping Responses (Responding by …):   - avoiding social interactions or places - seeking reassurance or expressing a desire for connection - self-isolation   Bodily Sensations (Experiencing …):   - physical symptoms of anxiety or panic - sensations of emptiness or numbness |
| --- |

Textbox S16. Features of Subjugation schema identified by GPT-4 (Textbox 1) and group of randomly sampled sentences for the schema.

| Schema Triggers (Situation where …):   - one feels compelled to suppress their true feelings to maintain harmony or avoid confrontation - one’s actions are driven by the anticipation of conflict or negative judgment   Emotions (Feeling …):   - anger or frustration in response to others’ actions - fear or anxiety about potential negative outcomes or social judgment   Negative Thoughts (Thinking that …):   - one lacks control over their life due to external factors - one’s actions or existence is a burden to others   Coping Responses (Responding by …):   - avoiding direct confrontation and expressing feelings indirectly - yielding to others’ demands to prevent conflict or ensure their happiness   Bodily Sensations (Experiencing …):   - - |
| --- |

Textbox S17. Features of Unrelenting Standards/Hyper-criticalness schema identified by GPT-4 (Textbox 1) and group of randomly sampled sentences for the schema.

| Schema Triggers (Situation where …):   - high expectations are placed by self or others - there is a need to maintain control or order   Emotions (Feeling …):   - inadequate or not good enough - overwhelmed by demands or pressure   Negative Thoughts (Thinking that …):   - one cannot cope or handle the situation - one must meet certain standards to be worthy   Coping Responses (Responding by …):   - internalizing feelings and self-blame - working excessively or pushing oneself too hard   Bodily Sensations (Experiencing …):   - fatigue or exhaustion - physical symptoms due to stress or anxiety |
| --- |

Textbox S18. Features of Vulnerability to Harm or Illness schema identified by GPT-4 (Textbox 1) and group of randomly sampled sentences for the schema.

| Schema Triggers (Situation where …):   - one anticipates or experiences a panic attack - one fears a negative health outcome - one is faced with social interactions or public places - one perceives everything is falling apart   Emotions (Feeling …):   - anxious or worried - overwhelmed or out of control - terrified or scared   Negative Thoughts (Thinking that …):   - one is going crazy or losing their mind - one’s health is in serious danger - something bad is going to happen   Coping Responses (Responding by …):   - avoiding situations or places - seeking reassurance or help   Bodily Sensations (Experiencing …):   - physical discomfort or pain related to anxiety - symptoms of panic such as heavy breathing or heart palpitations |
| --- |
